# Converging Neoplasms and the Environmental Exposome: Four Distinct Encounters with Collision Tumors in Dermatology

**DOI:** 10.7759/cureus.68031

**Published:** 2024-08-28

**Authors:** Jessica Forbes Kaprive, Stephanie Washburn, Alexandra Loperfito, Craig Garofola

**Affiliations:** 1 Dermatology, LewisGale Medical Center, Blacksburg, USA; 2 Dermatology, Edward Via College of Osteopathic Medicine, Blacksburg, USA; 3 Dermatology, River Ridge Dermatology, Blacksburg, USA

**Keywords:** sunburn, tobacco, mustard gas, squamous cell carcinoma, basal cell carcinoma, environmental exposure, collision tumor

## Abstract

Collision tumors - characterized by two or more distinct cell types within a singular lesion - are uncommon yet intriguing dermatological phenomena, presenting diagnostic and therapeutic enigmas. Our case series details four diverse presentations of such tumor intersections in dermatology. Beyond the individual cases, we embark on an exploration into the potential environmental exposome's role in the emergence of these neoplastic overlaps.

While the first and fourth cases underscore serendipitous discoveries during an excisional biopsy, the second revolves around diagnostic ambiguity arising from concurrent neoplasms. The third case delineates the challenges in surgical management due to intertwined tumor entities. Integral to our investigation, histopathological evaluations helped demarcate the distinct tumor types. We then delve into environmental factors - cumulative ultraviolet radiation, air pollutants, chemical carcinogens, and smoking - speculating their role in tandem neoplastic presentations.

Cutaneous collision tumors are infrequently occurring neoplasms of unknown origin characterized by two or more distinct cell types within a singular lesion. This series highlights a potential connection between specific environmental exposome and the development of collision neoplasms. An appreciation of this potential relationship will hopefully incite interdisciplinary collaborations and holistic management strategies, improving patient outcomes in the face of these dermatological rarities.

## Introduction

Collision tumors are characterized by two or more distinct cell types occurring within a singular lesion, with an estimated prevalence of around 69 per 40,000 cutaneous biopsies [1,2]. The most common combination observed occurs with basal cell carcinoma (BCC) and melanocytic nevus [1]. The pathogenesis for the development of collision remains unknown, though the most widely accepted hypothesis involves neoplastic heterogeneity [1]. Other proposed theories include the field cancerization theory and interaction theory, which suggest that recurrent skin damage increases the risk for the development of two neoplasms within one location and that the formation of one neoplasm produces epidermal or stromal changes inducing the formation of a second neoplasm, respectively [1]. Diagnosing these lesions requires a dermatoscopic exam and pathologic evaluation of the entire lesion to reduce the chance of overlooking a secondary tumor [1].

Treatment often involves excision, with margins based on the NCI guidelines for the more aggressive tumor [1]. The tumors involved often dictate prognosis, though the average disease-free interval for collision tumors is 22.7 months, suggesting they tend to be less aggressive than an individual neoplasm [1]. Here, we present a series of collision tumors and novel patient histories that highlight a potential connection between environmental exposomes and the development of collision neoplasms.

## Case presentation

Case one

**Figure 1:**
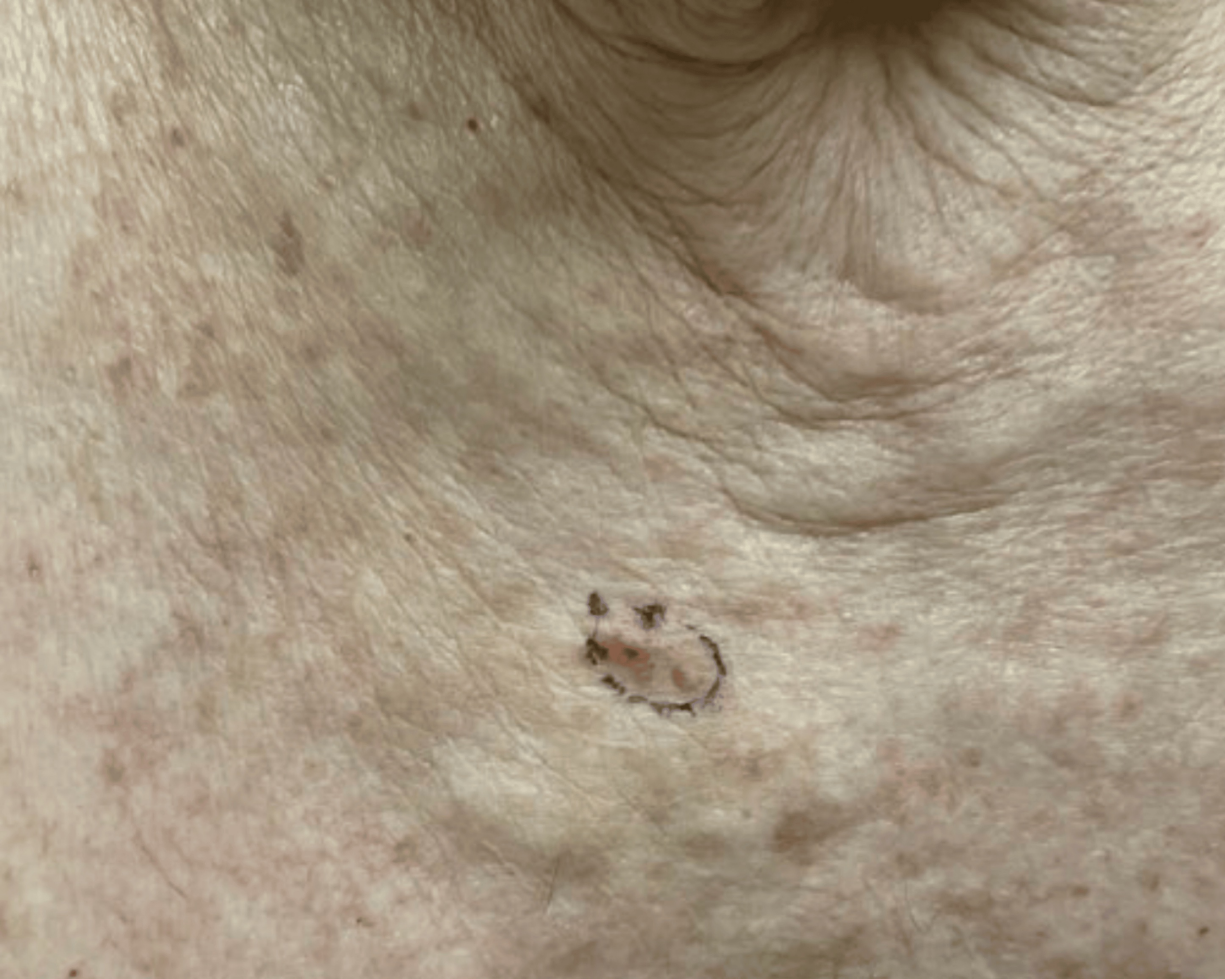
Melanoma-in-situ, focal basal cell carcinoma, and incidental seborrheic keratosis A 1.3 x 1.3 cm pink-brown papule located on the right medial superior chest was biopsied by shave method, resulting in Melanoma-in-situ, focal basal cell carcinoma, and incidental seborrheic keratosis.

**Figure 2:**
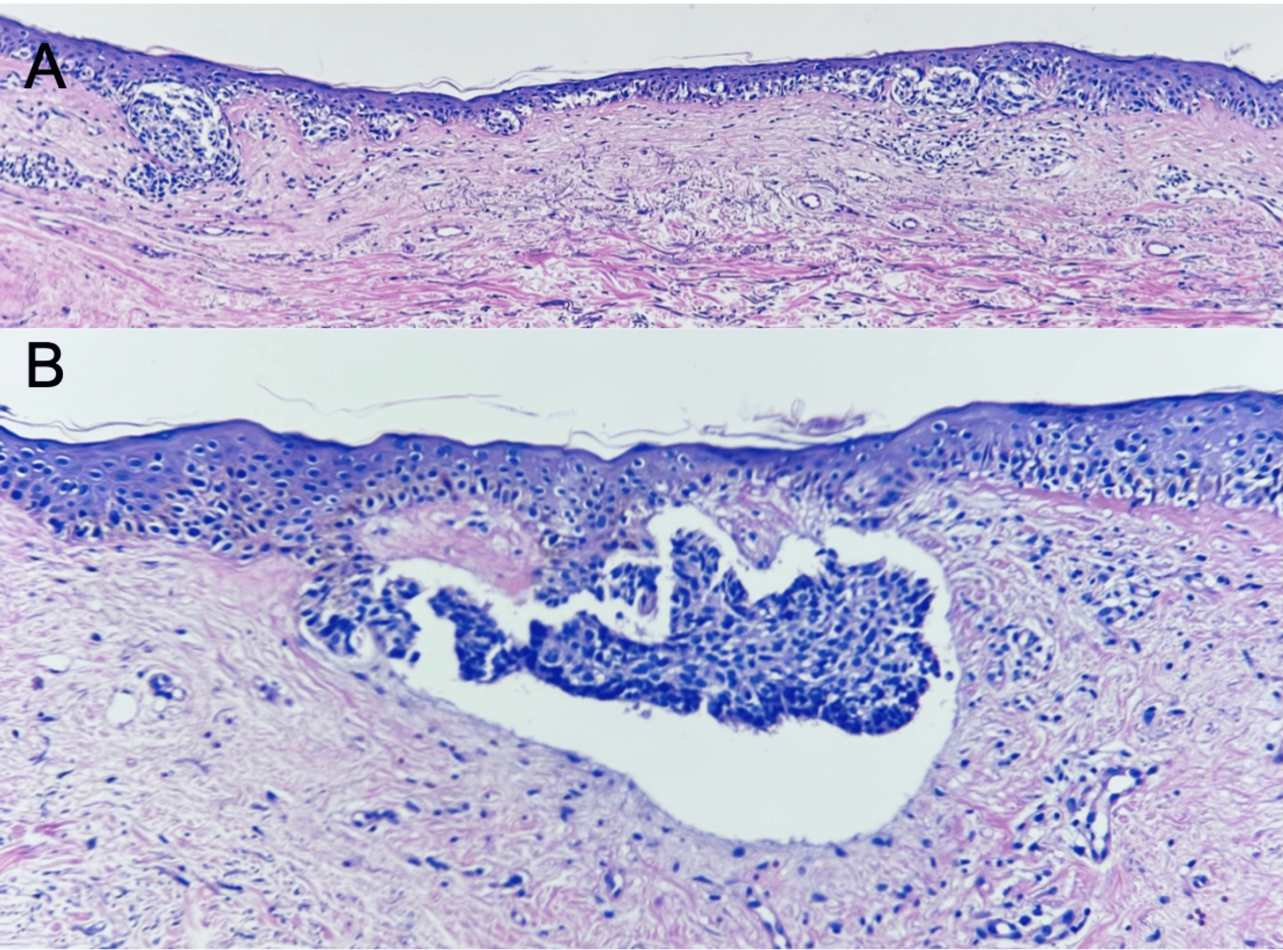
Histopathology of melanoma-in-situ, focal basal cell carcinoma, and incidental seborrheic keratosis (A) Moderately sized, poorly circumscribed focal proliferation of atypical melanocytes along the dermo-epidermal junction with pagetoid spread in the spinous layer indicated melanoma in situ. (B) Incidental superficial basal cell carcinoma location within the specimen.

The first case highlights a 91-year-old male with Fitzpatrick type II skin who presented for excision of biopsy-proven melanoma in situ with focal basal cell carcinoma and incidental seborrheic keratosis on the right medial superior chest (Figure 1). The neoplasm was treated with elliptical excision and complex repair (Figure 2). This patient had known exposure to mustard gas and cigarette smoke. Due to extensive third-degree burns sustained decades prior, he had a history of multiple plastic surgeries with autografting of full-thickness skin grafts. Our patient’s unique history of significant trauma following exposure to the toxic chemical sulfur mustard, also known as mustard gas, and later development of collision neoplasm highlights a possible connection to the role of the environmental exposome and the development of this peculiar multifaceted lesion.

Case Two

**Figure 3:**
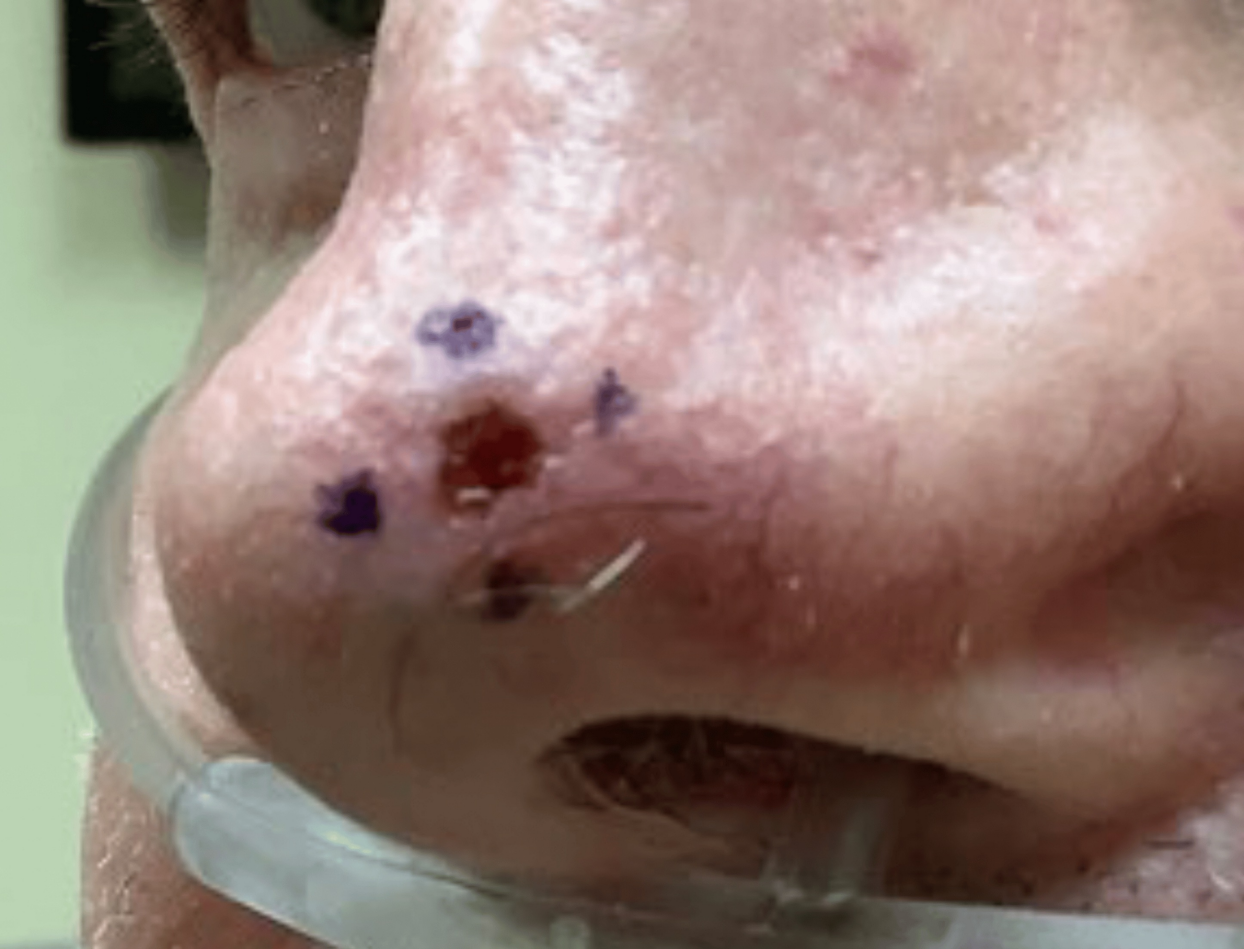
Nodular basal cell carcinoma and squamous cell carcinoma in situ A 0.5 x 0.5 ulcerated papule on the nasal tip which was biopsied by shave method resulting in nodular basal cell carcinoma. Later, during Mohs micrographic surgery, incidental squamous cell carcinoma in situ was recognized.

**Figure 4:**
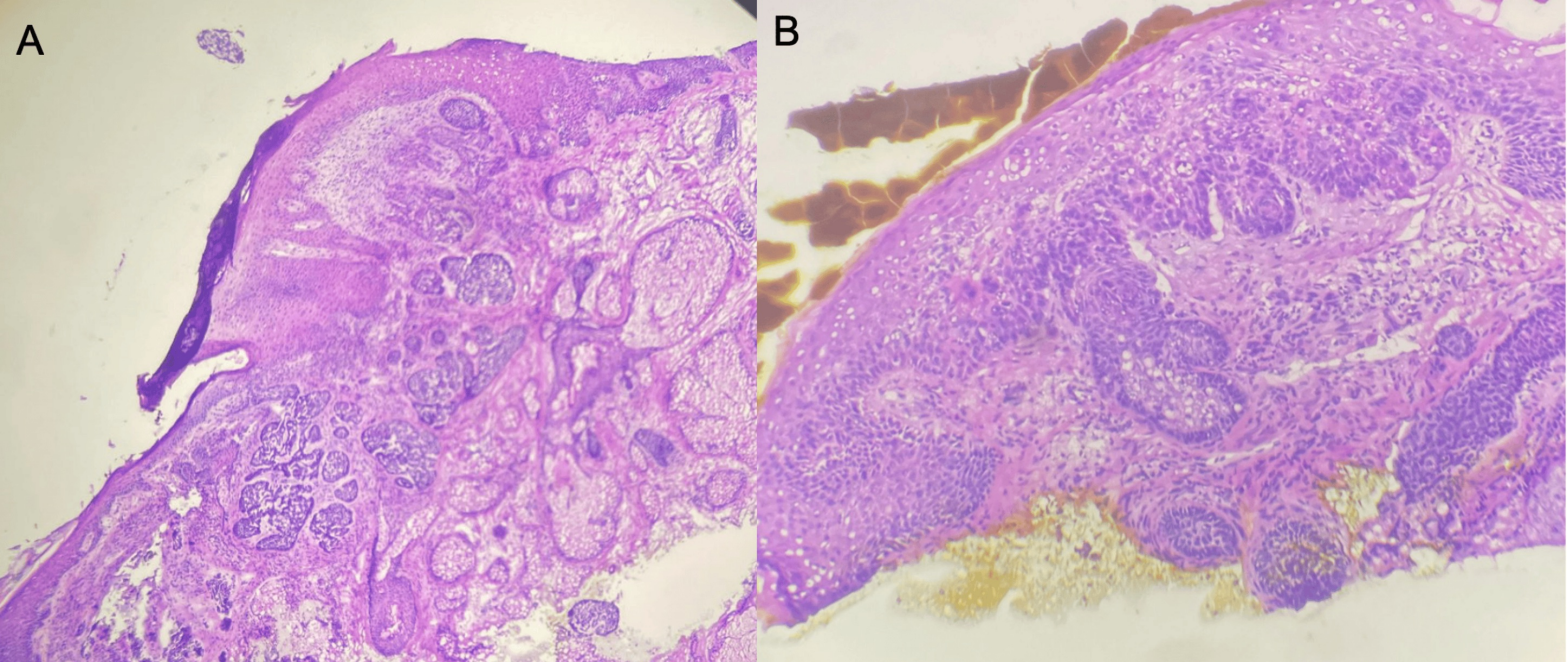
Histopathology of nodular basal cell carcinoma and squamous cell carcinoma in situ (A) Superficial papillary dermal islands of basaloid appearing cells with fibro-mucinous stroma indicating an infiltrative basal cell carcinoma. (B) Focal area of squamous cell carcinoma in situ located adjacently.

In the second case, we highlight a 64-year-old male patient with Fitzpatrick type II skin type and twenty-pack-years of smoking with a history of melanoma, squamous cell carcinoma (SCC), and basal cell carcinoma (BCC). A shave biopsy of an ulcerated papule on the nasal tip yielded a 0.7 cm x 0.9 cm nodular basal cell carcinoma (Figure 3). During subsequent Mohs micrographic surgery and pathologic analysis, we discovered a co-existing incidental squamous cell carcinoma in situ (Figure 4). During the operative and pathologic process of tumor clearance, the tumor required two stages for clearance and was repaired by utilizing a bilobed transposition flap. Of note, this patient had a previous longstanding history of tobacco use.

Case three

**Figure 5:**
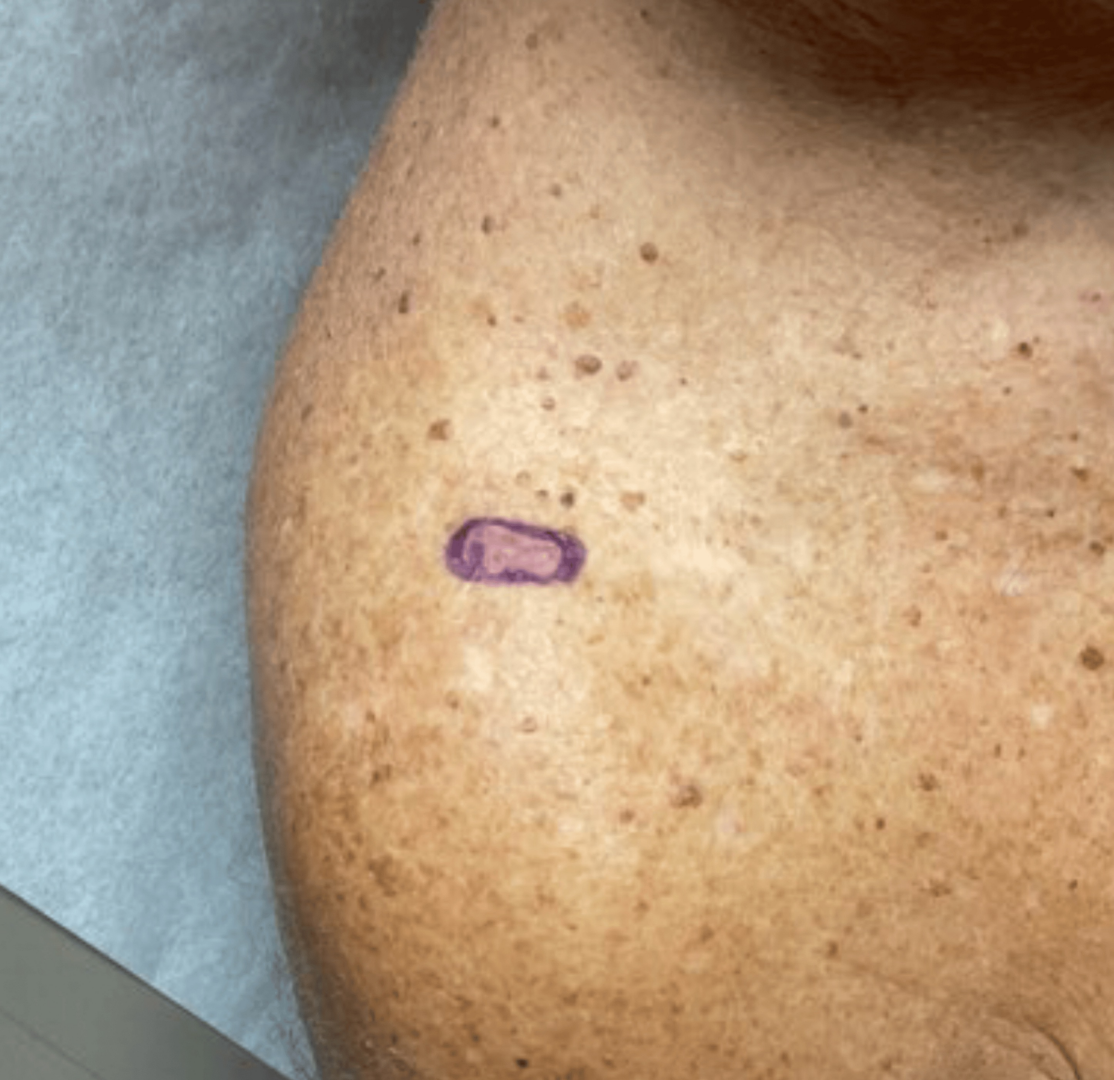
Superficial basal cell carcinoma with adjacent squamous cell carcinoma in situ A 1.4 x 0.6 cm pink pearly patch located on the right anterior shoulder

**Figure 6:**
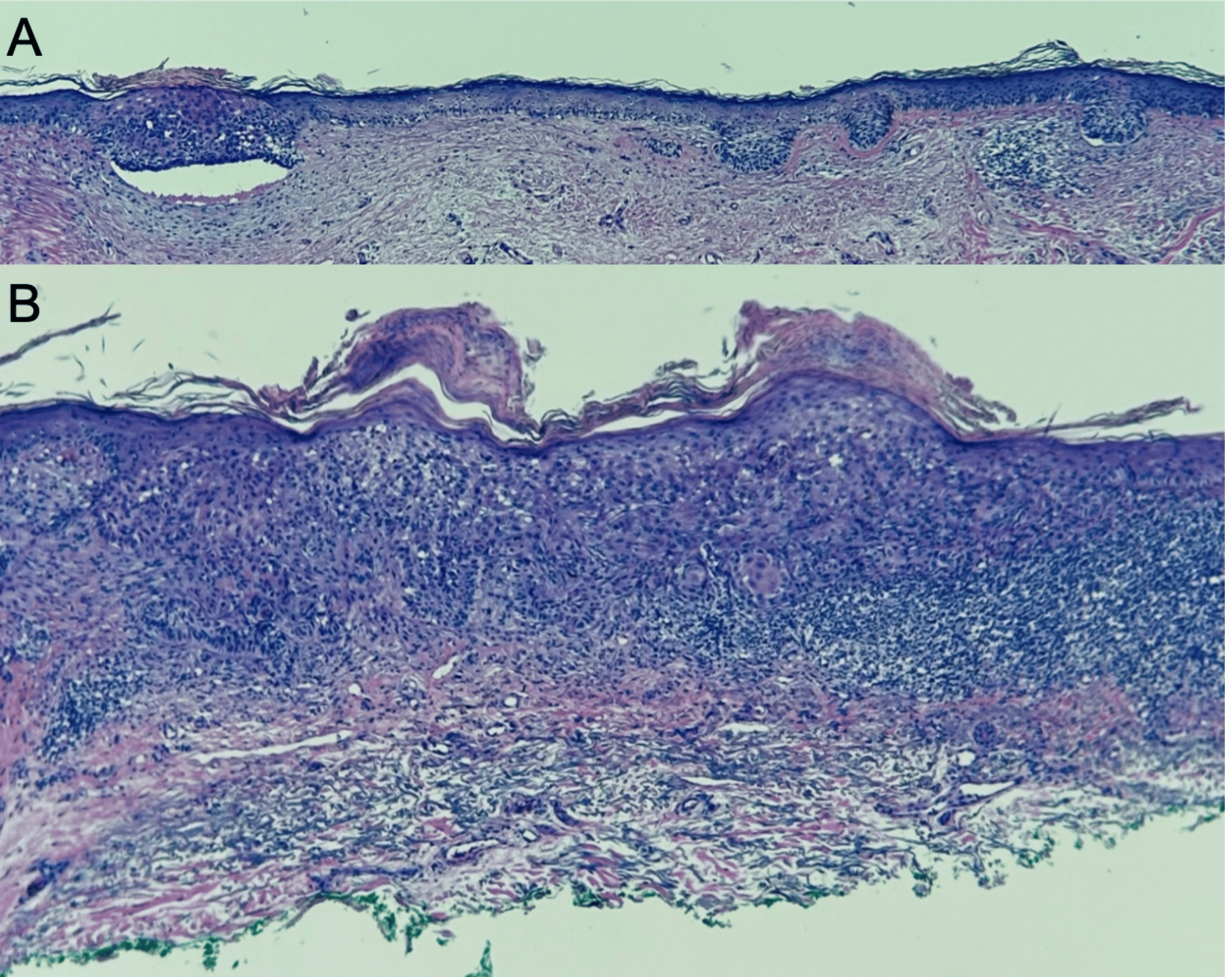
Histopathology of superficial basal cell carcinoma with adjacent squamous cell carcinoma in situ (A) Superficial, multifocal basal cell carcinoma with adjacent squamous cell carcinoma in situ (B) featuring full thickness squamous atypia, parakeratosis, and superficial lymphocytic inflammation.

The third case highlights a 75-year-old male with a history of multiple basal cell carcinomas who presented with a 1.4 cm x 0.6 cm pink pearly patch located on the right anterior shoulder (Figure 5). After a shave biopsy, this lesion yielded a superficial basal cell carcinoma with adjacent squamous cell carcinoma in situ (Figure 6). Notably, he also had a history of renal cell carcinoma (in remission).

Case four

**Figure 7:**
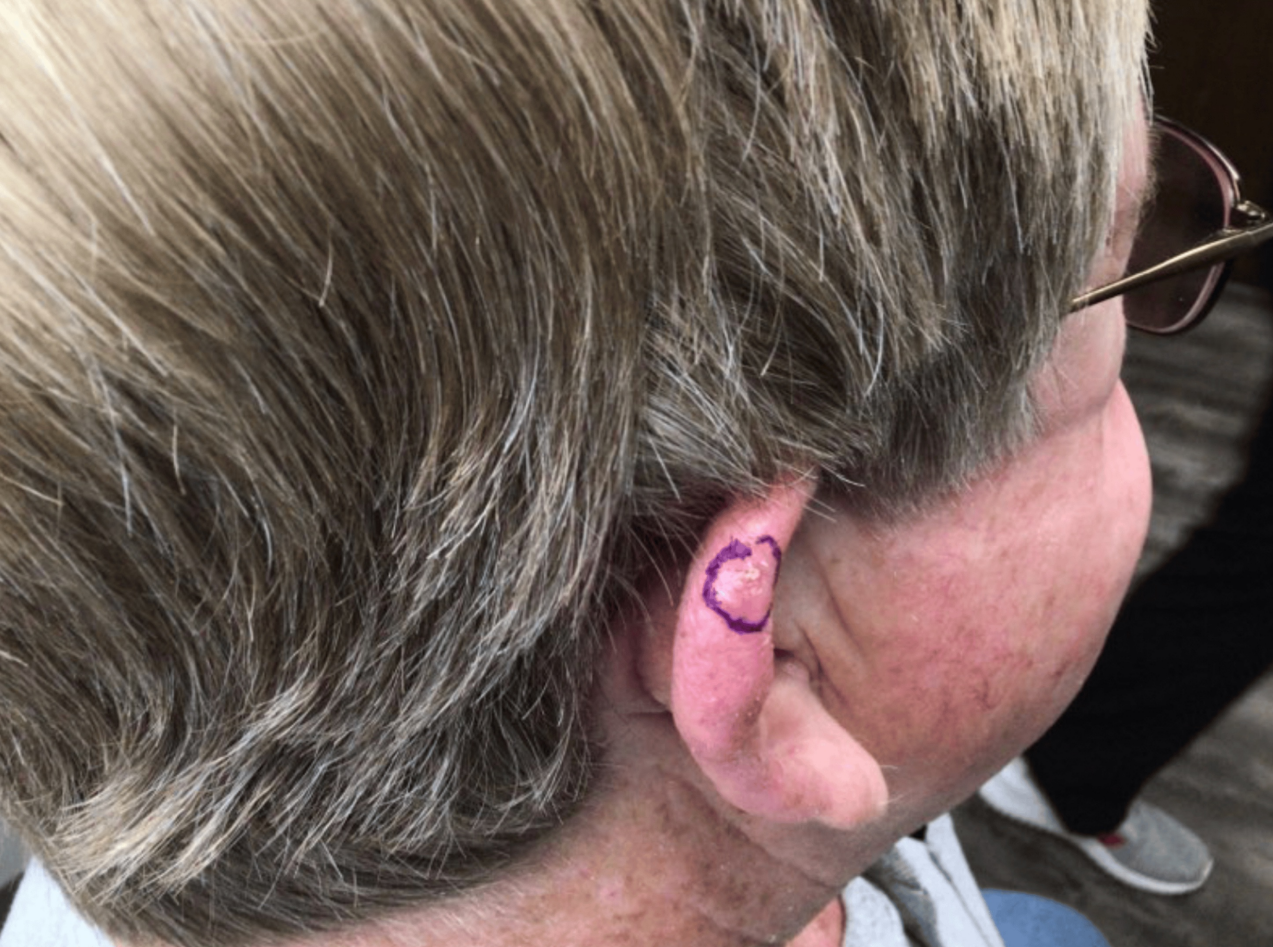
Atypical fibroxanthoma with incidental basal cell carcinoma and squamous cell carcinoma in situ An 0.7 x 0.5 cm ulcerative crusted papule on the superior helix which was biopsied and diagnosed as a 0.7 x 0.5 cm atypical fibroxanthoma. During Mohs micrographic surgery excision, incidental basal cell carcinoma and squamous cell carcinoma in situ were recognized.

**Figure 8:**
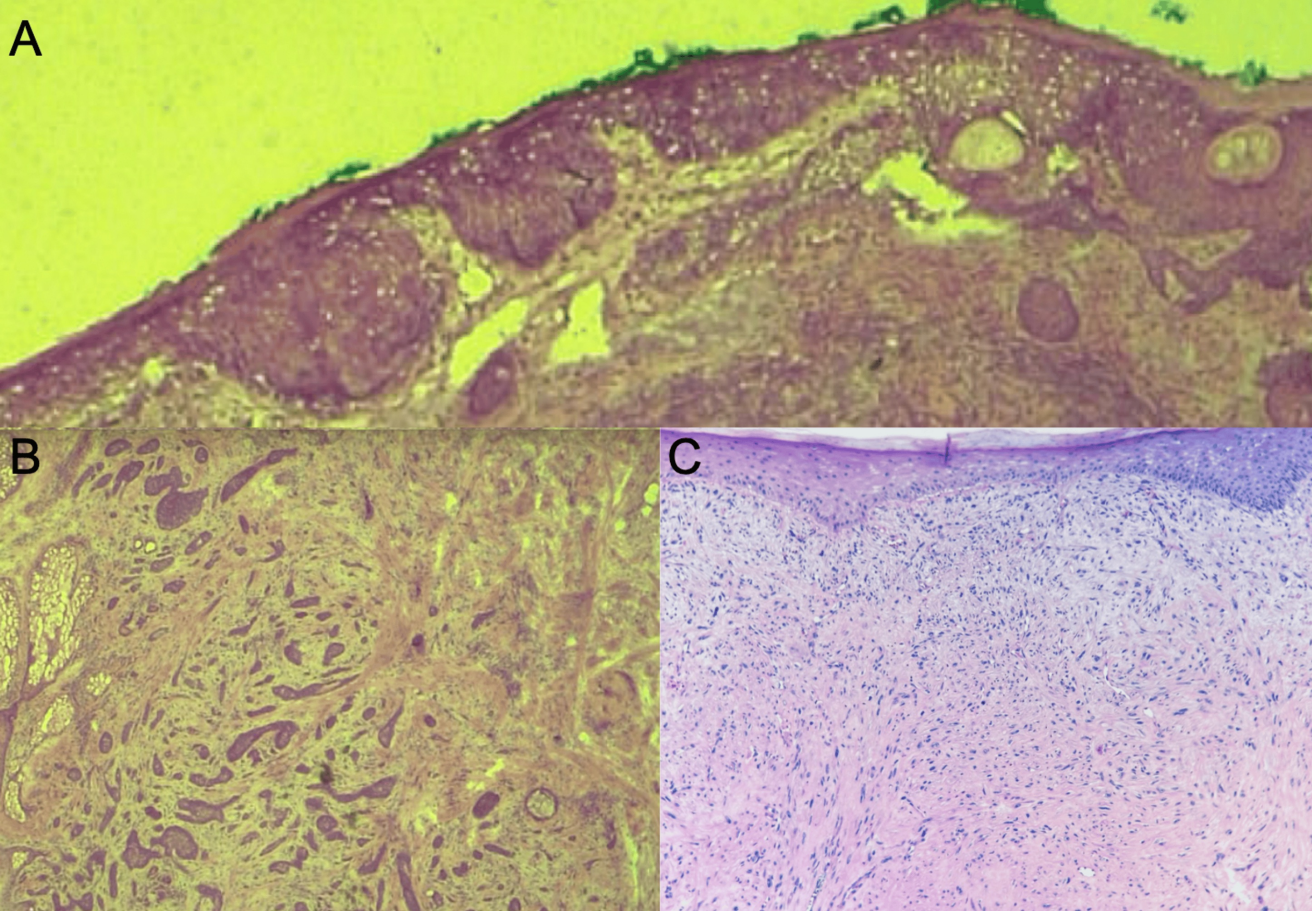
Histopathology of atypical fibroxanthoma with incidental basal cell carcinoma and squamous cell carcinoma in situ (A) Full thickness atypical keratinocytes, nuclear pleomorphism indicating squamous cell carcinoma in situ. (B) Thin islands of basaloid cells with nest-like configuration infiltrating between collagen fibers indicating infiltrative basal cell carcinoma. (C) Pleomorphic, spindled neoplasms which were immunoreactive for vimentin and CD10 and negative for melanocyte markers (S100, Melan-A), epithelial markers (multicytokeratin, p40), vascular marker (CD31), or fibrosarcomatous marker (CD34) revealing a diagnosis of exclusion, atypical fibroxanthoma.

The fourth case highlights a 69-year-old Caucasian male patient with Fitzpatrick type II skin type and a history of multiple prior BCC and SCC. This patient developed an ulcerative, crusted papule on the superior helix (Figure 7). The lesion was biopsied and diagnosed as a 0.7 cm x 0.5 cm atypical fibroxanthoma. During Mohs micrographic surgery and pathologic analysis of two stages to remove the tumor, an incidental BCC and squamous cell carcinoma in situ (SCCis) were found within the tumor (Figure 8). This patient reports an extensive history of blistering sunburns in the past and no other pertinent history of smoking or environmental exposures.

## Discussion

Exposome

Ultraviolet Radiation and Skin Phototype

Ultraviolet radiation has a well-established dose-dependent association with the development of non-melanoma skin cancer [3]. Studies have shown that Caucasians residing in regions close to the equator are more greatly affected by the association between UV exposure and increased skin cancer risk, with significantly more cases in both males and females living in Queensland, Australia compared to the United States [4]. Cutaneous melanin level is one of the most important factors affecting the risk of skin cancer development [5]. Melanin acts as a UV absorbent, with additional evidence for antioxidant and free radical scavenging properties [5]. Baseline skin eumelanin levels are inversely correlated with DNA damage, with increasing eumelanin levels showing more efficient repair of sun-induced DNA damage [6]. Skin phototypes I-II were affected with malignant melanoma (MM) and non-melanoma skin cancer (NMSC) in 59% and 54% of skin cancer cases [3]. Though the exact etiology of collision tumors remains unclear, UV damage, along with patient age, skin type, and reactive oxygen species, likely plays a role in their development [7].

Air Pollutants

Polycyclic aromatic hydrocarbons, particulate matter, and smoke are common air pollutants with detrimental effects on the skin by triggering an increase in oxidative stress and depleting antioxidant capacity [8]. Additionally, these pollutants can alter lipids, deoxyribonucleic acid, and proteins, resulting in extrinsic aging, inflammation, and allergic skin pathology [8]. Chronic polycyclic aromatic hydrocarbon (PAH) exposure may activate the aryl hydrocarbon receptor (AhR) transcription factor affecting epidermal targets, thus contributing to skin cancer development [9]. Particulate matter exposure has been associated with mitochondrial DNA oxidative injury [9]. It has been reported that exposure to a 10 µg/m3 concentration increase in particulate matter of ≤10 micrometers (PM10) may carry a 52% higher relative risk for non-melanoma skin cancer [9].

Occupational Exposures and Chemical Carcinogens

Exposure to carcinogens has been associated with the development of squamous cell carcinoma and melanoma, often in a stepwise fashion [10]. Exposure to alkylating agents and nitrosamines results in the induction of deoxyribonucleic acid damage, leading to genetic change [10]. These changes, in combination with repeated exposure to noncarcinogenic promoting agents, create an environment conducive to clonal outgrowth and premalignant tumor development, either spontaneously or with repeated exposure [10].

Mustard gas is an agent of chemical warfare shown to have catastrophic effects on the skin, pulmonary, and ocular systems of those exposed [11]. Due to the nucleophilic and lipophilic properties of sulfur mustard, it can easily diffuse throughout the body, altering DNA structure via an alkylation reaction promoting mutation and carcinogenesis [12]. Though initial presentation of skin findings resembles first- or second-degree burns and usually heals spontaneously in four to six weeks, there have been reports of the development of multiple SCCis in Japanese factory workers who were exposed to sulfur mustard in the workplace [13,14].

Occupational exposures to polycyclic aromatic hydrocarbons and ionizing radiation have also demonstrated a correlation with certain skin cancers [15]. Polycyclic aromatic hydrocarbon exposure occurs primarily through inhalation in the coal and gas industry, aluminum production, and steel foundries, and among those with exposure to diesel exhaust fumes [15]. Mechanisms for skin cancer causation have not been identified, but BCC and SCC appear at increasing frequencies in these populations [15]. Although limited in exposure today, the ionizing radiation exposure of pre-1950 radiological technologists working without lead aprons increased the incidence of SCC and MM [15].

Arsenic exposure is common in workers involved in the manufacturing of glass and semiconductors as well as in those exposed to insecticides and herbicides [15]. Studies have indicated that systemic absorption through ingestion is the most common route of exposure, with many resulting arsenical keratoses found in these individuals [15]. These pathognomonic lesions are primarily found on the palms and soles and are punctate. They typically appear as corn-yellow hyperkeratotic papules and plaques [15]. Though rare, case reports have shown progression to squamous cell carcinoma [15].

Smoking

There is a well-established relationship between cigarette smoking and many cancers. In recent years, smoking has been shown to increase the risk of skin cancer [16]. Studies have shown that current smokers have an increased risk of SCC when compared to never-smokers, but a slightly decreased risk of MM and BCC [16]. Former smokers, on the other hand, did not have an increased risk of SCC, BCC, or MM [16]. More research is needed into the recognized decreased risk of MM and BCC, but hypotheses have been proposed including nicotine-induced acceleration of skin elastosis protecting against melanoma, an interaction with genes conferring susceptibility to BCC, and the possibility of detection bias [16].

Review

Our case series highlights the clinical intricacies of collision tumors and environmental factors influencing their genesis. The co-occurrence of malignant neoplasms may not be a purely random event, as suggested by a retrospective study of 11 cases of contiguous malignant melanoma and basal cell carcinomas [1]. One proposed mechanism for the development of collision tumors, the field cancerization theory, suggests that recurrent skin damage increases the likelihood of two separate neoplasms in a singular location [1]. The increased number of collision tumors developing in areas affected by UV radiation, burns, and xeroderma pigmentosum supports this theory [1]. In our evaluation of four cases of collision tumors, the following exposures were noted: sulfur mustard, third-degree burns, tobacco use, and blistering sunburns. Each patient’s risk factors have known associations with skin cancer, but their uniqueness derives from the new associations with multiple cell types within a single lesion.

Collision tumors commonly involve one benign and one malignant lesion, most frequently a melanocytic nevus and BCC [1]. Tumors consisting of seborrheic keratosis and BCC have also been documented, with one study noting this collision pattern in eight of 69 cases [2,17]. In this case series, one of the four cases included a BCC and seborrheic keratosis (SK). However, this case also included a melanoma in situ as part of the collision in a patient exposed to sulfur mustard.

Although less common, collision tumors involving atypical fibroxanthoma (AFX), BCCs, and SCCs have been reported [18]. Atypical fibroxanthomas are generally considered benign with a good prognosis, but those with certain histopathological traits and extension into the subcutaneous tissue have a poorer prognosis [18]. The primary risk factor for AFX is UV radiation [18]. This exposome highlights a case of a collision tumor consisting of atypical fibroxanthoma, BCC, and SCCis in a patient with a long history of blistering sunburns and prolonged exposure to UV radiation.

Collision tumors comprising two malignant lesions have been scarcely reported, with a retrospective study of 78,000 primary cutaneous neoplasms reporting 11 cases of basal cell carcinoma and melanoma collision tumors [1,18]. A review of the literature by Cornejo and Deng notes 17 BCC and melanoma collision tumors in 51 cases [19]. One case in this exposome involved a melanoma in situ and BCC with a patient history significant for environmental exposure to mustard gas and a history of severe burns. The field cancerization theory suggests that recurrent skin damage from severe burns and chemicals may increase the likelihood of developing two neoplasms in one location [1].

Collision tumors composed of BCC and SCC have been infrequently reported. One case report describes a BCC and SCC in situ occurring on the palm in a 71-year-old female with no history of skin cancer, arsenic exposure, burn to the area, or ionizing radiation exposure [20]. In our case series, three of four patients were noted to have a BCC and SCC collision tumor. These cases occurred in sun-exposed areas and the patients had known exposures of blistering sunburn and extensive tobacco use.

## Conclusions

Cutaneous collision tumors are infrequently occurring neoplasms of unknown origin characterized by two or more distinct cell types within a singular lesion. Our retrospective case series highlights specific environmental exposures - mustard gas, third-degree burns, tobacco use, and blistering sunburns - and the development of cutaneous collision tumors. Though larger patient population studies are required to determine strong correlation data between exposure and cutaneous neoplasm development, this series highlights a potential connection between specific environmental exposome and the development of collision neoplasms. An appreciation of this potential relationship will hopefully incite interdisciplinary collaborations and holistic management strategies, thereby improving patient outcomes in the face of these dermatological rarities.
